# The shift from plant–plant facilitation to competition under severe water deficit is spatially explicit

**DOI:** 10.1002/ece3.2875

**Published:** 2017-03-12

**Authors:** Michael J. O'Brien, Francisco I. Pugnaire, Cristina Armas, Susana Rodríguez‐Echeverría, Christian Schöb

**Affiliations:** ^1^ Estación Experimental de Zonas ÁridasConsejo Superior de Investigaciones CientíficasAlmeríaSpain; ^2^ Centre for Functional EcologyDepartment of Life SciencesUniversity of CoimbraCoimbraPortugal; ^3^ Department of Evolutionary Biology and Environmental StudiesUniversity of ZurichZurichSwitzerland

**Keywords:** biodiversity, competition, plant community diversity, plant–climate interactions, spatial variability, stress‐gradient hypothesis, water limitation

## Abstract

The stress‐gradient hypothesis predicts a higher frequency of facilitative interactions as resource limitation increases. Under severe resource limitation, it has been suggested that facilitation may revert to competition, and identifying the presence as well as determining the magnitude of this shift is important for predicting the effect of climate change on biodiversity and plant community dynamics. In this study, we perform a meta‐analysis to compare temporal differences of species diversity and productivity under a nurse plant (*Retama sphaerocarpa*) with varying annual rainfall quantity to test the effect of water limitation on facilitation. Furthermore, we assess spatial differences in the herbaceous community under nurse plants *in situ* during a year with below‐average rainfall. We found evidence that severe rainfall deficit reduced species diversity and plant productivity under nurse plants relative to open areas. Our results indicate that the switch from facilitation to competition in response to rainfall quantity is nonlinear. The magnitude of this switch depended on the aspect around the nurse plant. Hotter south aspects under nurse plants resulted in negative effects on beneficiary species, while the north aspect still showed facilitation. Combined, these results emphasize the importance of spatial heterogeneity under nurse plants for mediating species loss under reduced precipitation, as predicted by future climate change scenarios. However, the decreased water availability expected under climate change will likely reduce overall facilitation and limit the role of nurse plants as refugia, amplifying biodiversity loss.

## 1 Introduction

An enduring theory in ecology, the stress‐gradient hypothesis (Bertness & Callaway, 1994), provides a framework for positing the outcome of interactions among organisms along a gradient of environmental conditions (Bakker, Dobrescu, Straile, & Holmgren, 2013; Fugère et al., 2012; Pugnaire & Luque, 2001). This theory predicts that competitive interactions are more common in more productive environments, but as environmental stress and disturbance increases (e.g., reduced nutrient or water availability, extremely low temperatures, and high consumer pressure), competition gives way to facilitation (Bertness & Callaway, 1994; Brooker & Callaghan, 1998; He, Bertness, & Altieri, 2013). Refinements to this theory suggested that at the furthest extreme of resource limitation facilitation will decrease (Michalet et al., 2006) or even revert to competition (Michalet, Le Bagousse‐Pinguet, Maalouf, & Lortie, 2014), in particular if competitive rather than stress‐tolerant species and consumers are involved (Choler, Michalet, & Callaway, 2001; Maestre, Callaway, Valladares, & Lortie, 2009; Smit, Rietkerk, & Wassen, 2009). However, direct evidence regarding changes in sign and intensity of plant–plant interactions under changing resource conditions, including cases of severe resource limitation, is important for understanding the response of species and communities to a changing climate (Brooker et al., 2008; Butterfield, Betancourt, Turner, & Briggs, 2010).

Whether the outcome of plant–plant interactions is competitive, facilitative or neutral depends on many factors including environmental conditions (e.g., rainfall and soil nutrient availability), the identity of the coexisting species, and the ontogeny and the functional type of the interacting species (Armas & Pugnaire, 2005; Armas, Rodríguez‐Echeverría, & Pugnaire, 2011; Espeland & Rice, 2007; Maestre et al., 2009; Pugnaire, Armas, & Maestre, 2011; Tielbörger & Kadmon, 2000). Furthermore, facilitation by a nurse species may not be symmetric (López‐Pintor, Gómez Sal, & Rey Benayas, 2006; Pugnaire, Luque, Armas, & Gutiérrez, 2006). For example, competitive effects may increase on southern aspects of the nurse plant, while facilitative effects persist on a northern aspect due to differences in temperature and light that alter evapotranspiration (López‐Pintor et al., 2006). Therefore, knowledge about the spatial variability at the microsite level in nurse plant systems is important to assess the cumulative effects of plant–plant interactions (Butterfield, 2009; Butterfield, Bradford, Armas, Prieto, & Pugnaire, 2016; Michalet, Brooker, Lortie, Maalouf, & Pugnaire, 2015). However, spatial variability may also mediate the negative effects of severe resource limitation.

Plant–plant interactions often supersede the effects of climate change by exacerbating competition or driving novel competitive interactions (Alexander, Diez, & Levine, 2015; Liancourt et al., 2013), but interactions among plants may also alleviate extreme climatic conditions (López, Squeo, Armas, Kelt, & Gutiérrez, 2016). Indeed, facilitation is often observed in systems that are experiencing growth‐ or fitness‐limiting climatic conditions, such as hot and dry semiarid ecosystems or savannas (Butterfield et al., 2016; Dohn et al., 2013; Pugnaire et al., 2011). Therefore, facilitative interactions may promote species diversity by maintaining species in conditions outside their fundamental niche (Bruno, Stachowicz, & Bertness, 2003). This greater species diversity is important for ecosystem functions such as productivity and ecosystem stability (Hector et al., 1999; Isbell et al., 2015; Tilman, Reich, & Knops, 2006). This stabilizing effect may be especially important for semiarid ecosystems that are already experiencing important climatic changes, which are pushing the environmental conditions to novel extremes (Giorgi & Lionello, 2008; Lloret, Siscart, & Dalmases, 2004). As an example, the yearly rainfall of the Mediterranean Basin is expected to decrease as much as 20% within the next 50 years, while temperatures are likely to increase (IPCC 2014). In conjunction with these gradual changes, extreme events such as long heat waves and heavy precipitation events are predicted (Lloret et al., 2004). Combined, these climatic shifts have potentially important consequences for the currently prevailing facilitative interactions found in semiarid systems and the biodiversity and ecosystem functions that depend on those positive plant–plant interactions (Brooker et al., 2008; Mueller et al., 2005).

In this study, we compare the relative effect of nurse plants on species diversity (richness) and community productivity (plant biomass) of vascular plants in a range of years varying from low to high rainfall (Table 1) to assess nurse plant effects on herbaceous communities across a range of water availability. We also tested spatial differences in diversity, productivity, abundance, and composition of the herbaceous plant communities in the four cardinal directions around nurse plants during a year with severely low rainfall. This detailed spatial assessment was carried out under a species (*Retama sphaerocarpa*) that has been well documented to facilitate diversity at this site under average climatic conditions (Armas et al., 2011; Pugnaire & Lázaro, 2000; Pugnaire et al., 1996). Our hypothesis was that severe water limitation promoted negative plant–plant interactions but the magnitude of that effect depended on variation in microsite conditions under the nurse species.

**Table 1:** Environmental variables measured near the sampling location in Tabernas, Spain. The means were calculated for the period from October to March for all years between 2000 and 2016. The period between October 2011 and March 2012—the sampling year—had below‐average rainfall, average maximum daily temperature, and above‐average radiation

Environmental variable	Mean (95% CI)	2008/2009	2009/2010	2011/2012	2014/2015
Cumulative precipitation (mm)	164 (123–206)	201	351	65	129
Maximum daily temperature (°C)	18.1 (17.7–18.5)	16.3	19.5	18.0	18.0
Daily radiation (MJ m^−2^)	12.4 (12.1–12.6)	12.1	11.7	13.3	12.7

## 2 Materials and methods

### 2.1 Study site

**Figure 1:**
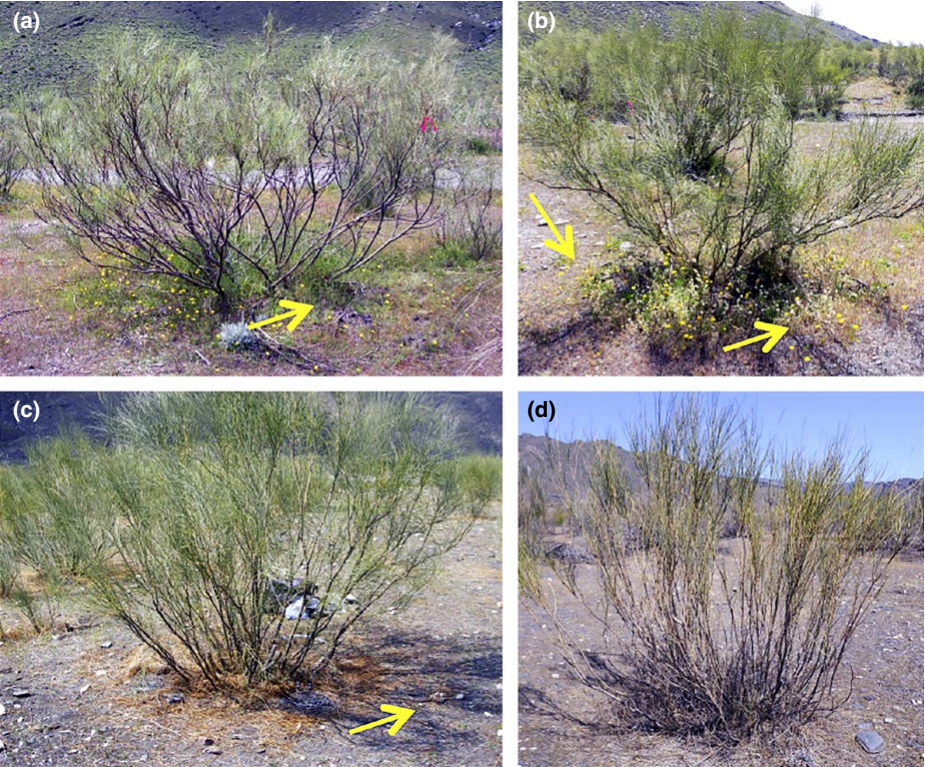
Plant communities in four different years of rainfall. (a) The plant community around *Retama* in March 2010 after above‐average rainfall (~351 mm). (b) The plant community around *Retama* in early April 2011 with average rainfall (~178 mm). (c) The plant community around *Retama* in March 2012 (the sampling year) with below‐average rainfall (~65 mm). (d) The plant community around *Retama* in March 2016 with below‐average rainfall (~73 mm). The yellow arrows indicate the presence of annual plants

Fieldwork was conducted in a *Retama sphaerocarpa* shrubland located in Rambla del Saltador, Almería Province, SE Spain (37°08′N, 2°22′W) at 630 m elevation. Climatic conditions are semiarid with a dry summer season between June and September. Mean annual rainfall is ~250 mm and annual mean temperature is 16°C (Pugnaire & Lázaro, 2000). The soil at the valley bottom is a sandy loam of alluvial origin with poor water‐holding capacity and low soil organic matter and nutrient concentrations (Pugnaire et al., 1996). The sparse vegetation at the valley bottom is dominated by *R. sphaerocarpa*, a leguminous shrub with very deep roots known to be involved in the redistribution of water in the soil (Prieto, Kikvidze, & Pugnaire, 2010). These shrubs are distributed homogenously throughout the area and surrounded by large open areas (Figure 1). Both the shrub understory and the surrounding open areas are mainly colonized by annual grasses and herbs, but the vegetation is generally denser and more species‐rich below shrubs than in open areas (Pugnaire et al., 1996). This generally facilitative effect of the shrub is attributed to higher water availability (Prieto et al., 2010; Pugnaire, Armas, & Valladares, 2004) as well as higher soil organic matter and nutrient concentration (Pugnaire et al., 1996, 2004) along with ameliorated environmental conditions such as lower air and soil temperatures (Moro, Pugnaire, Haase, & Puigdefábregas, 1997; Pugnaire et al., 2004).

### 2.2 Sampling design

In March 2012, we sampled seven *Retama* shrubs and seven neighboring open areas (hereafter referred to as *Retama* and open locations). For each *Retama* plant, we sampled four plots, one at each cardinal direction: north, east, south, and west. These four plots were paired with plots in the open locations about 4 m outside the canopy in the same four directions. Each circular plot had a radius of 10.6 cm (353 cm^2^) with a standardized sampling grid divided into three concentric circles. The innermost circle was further divided into three sections, and the two outer circles were divided into six sections (see Fig. S1 in Supporting Information). For each plot, we recorded the number of individuals of each vascular plant species (i.e., richness and total plant abundance) and their specific location within the sampling grid (Fig. S1). For each individual (486 individuals in total), we collected the largest healthy leaf (including the petiole) and measured dry biomass (total leaf biomass).

### 2.3 Abiotic conditions

We measured photosynthetically active radiation (PAR), soil temperature, and soil depth within each part of the sampling grid. PAR was measured 10 cm above ground with a LS‐C Mini Quantum Sensor (Heinz Walz GmbH, Germany). Soil temperature was measured at 2 cm below the soil surface with handheld thermometers. Soil depth was measured with a penetrometer that was inserted into the soil until it could not be introduced further without reasonable effort. Soil depth was measured as the length of the penetrometer that was inserted into the soil. Plots in open locations were always measured immediately after the associated plot beneath *Retama* in the same aspect to reduce temporal differences in temperature and light within each pair of *Retama* and open plots (see Table S1 in Supporting Information).

### 2.4 Interannual facilitation data

We compiled data on herbaceous communities under *Retama* and in open locations from studies performed in the same geographic area as our 2012 sampling (Armas et al., 2011; Hortal et al., 2015; Schöb, Armas, & Pugnaire, 2013). The sampling design of these studies was comparable to our detailed spatial assessment in 2012 with sampling occurring in late March or early April in 400 cm^2^ plots (similar in size to the 353 cm^2^ plots in 2012). Plots were located in the west in 2009 (Armas et al., 2011), the north in 2010 (Schöb et al., 2013) and in all aspects around *Retama* in 2015 (Hortal et al., 2015). We used these data to perform a meta‐analysis comparing facilitative interactions across different years with different quantities of rainfall. Our assessment with these data does not overlap the outcomes of the original studies, which assessed facilitation across an aridity gradient (Armas et al., 2011), competition among beneficiary species (Schöb et al., 2013), and differential effects of benefactor and allelopathic shrubs on soil microbes (Hortal et al., 2015).

### 2.5 Statistical analysis

In order to test the hypothesis that facilitative effects of *Retama* gave way to competitive effects under severely low rainfall, we calculated the relative interaction intensity (RII; Armas, Ordiales, & Pugnaire, 2004) for species richness (i.e., diversity) and plant biomass (i.e., productivity) for herbaceous communities under *Retama* from our sampling in 2012 as well as from the sampling that was carried out in the same area during the three additional years (Table 1). We analyzed RII as a function of total rainfall (measured at a weather station located at the site; http://www.juntadeandalucia.es/) from October to March (a continuous variable) with a linear mixed‐effects model. We also used an *a priori* linear contrast with total rainfall as a factor to test whether RII responded nonlinearly to rainfall. Sample (individual plots under *Retama*) nested within year was used as a random effect, and we accounted for heteroscedasticity with a separate variance structure for each year. Because plots under *Retama* and in open locations were not paired in every study (other studies randomly assigned plots in open locations), RII was calculated as the difference between each herbaceous community under *Retama* and the mean values of the open plots from that sampling year (i.e., mean of open locations from each year for each sampling) divided by the sum of those two variables. Community‐level biomass was not collected in 2012. Therefore, we used the dry biomass of the largest leaf from each individual aggregated by plot as a proxy for total biomass (which correlates well with total plant biomass; see Fig. S2 in Supporting Information). However, different productivity metrics are controlled for in the calculation of RII because all observations are relative to the average value of open plots in the same sampling year. Positive RII values indicate facilitative effects, while negative RII values may suggest competitive effects or biological filtering (e.g., interference). Furthermore, we assume that an increase in community diversity and productivity indicates a facilitative effect on the species that comprise that community. For example, higher species richness under a nurse plant means that species exist under the nurse plant that do not exist in open space—obligatory facilitation (Bertness & Callaway, 1994)—or that more species from the overall species pool exist under the nurse plant.

To test the hypothesis that facilitative effects under *Retama* are spatially dependent during a drought, we analyzed species richness, total leaf biomass (sum of the dry biomass for all the largest leaves in a plot), and plant abundance from the 2012 sampling as a function of location (a fixed factor with two levels: *Retama* and open), aspect (a fixed factor with four levels: north, east, south, and west), and their interaction. We accounted for differences in plot conditions with covariates for mean soil depth and temperature. Light and soil temperature were highly correlated (r = .45, *p *=* *.0005), so only temperature was used in the models, but the inclusion of light as a covariate did not affect the main results. Random effects were included for *Retama* plant (seven levels), location nested within *Retama* plant (14 levels), and aspect nested within *Retama* plant (28 levels). To meet assumptions of normality, we used a separate variance structure for location in the analysis of species richness, and we log‐transformed biomass plus 1 for the total leaf biomass (abundance met assumptions of normality).

Dissimilarity among plant community composition was calculated on the presence–absence of all plant species using the Jaccard index. We used presence–absence because of the number of plots with no species or only one species. The plant community dissimilarity was compared among plots in all four aspects both under and outside each *Retama*. The effect of the presence of *Retama* on plant community composition was assessed using a constrained analysis of principal coordinates with location (a factor with two levels), aspect (a factor with four levels), and their interaction as the constraining variables of the Jaccard dissimilarity matrix. Therefore, a significant effect of *Retama* on the community composition indicates that unique species with respect to the open plots are present due to facilitative effects (i.e., these species are only present due to facilitation by *Retama*).

Linear mixed‐effects models were performed with the asreml‐R package (ASReml 3, VSN International, UK) in the R statistical software (version 3.3.1; http://r-project.org). The Jaccard index was calculated with the vegdist function in the vegan package. The constrained analysis of principal coordinates was performed with the capscale function, and significance was tested with a permutation‐based ANOVA for each term with default settings.

## 3 Results

**Figure 2:**
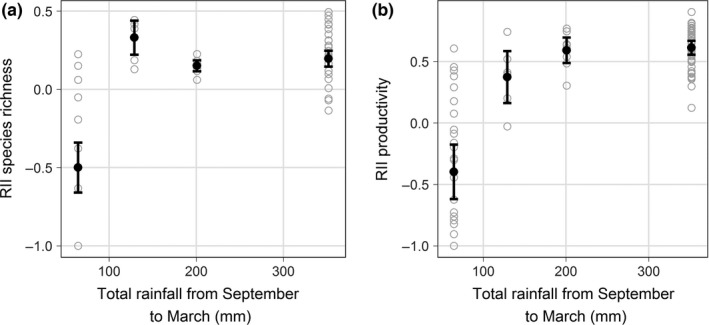
Relative effect of *Retama* on species richness and productivity. Relative interaction intensity for (a) plant species richness and (b) community productivity as a function of total rainfall from September to March from four different years of sampling near Tabernas, Spain. The black points represent model estimates (95% CI), and the gray points are individual samples. Three of the data sets (2009, 2010, and one from 2015) have been published (Armas et al., 2011; Hortal et al., 2015; Schöb et al., 2013)

Relative interaction intensity (RII) for both species richness and productivity shifted from positive (i.e., facilitation) to negative (i.e., competition) as total rainfall decreased. However, the significant categorical rainfall variable—fit after the continuous term (Table S2)—indicates that RII did not respond linearly to decreasing rainfall. Therefore, below a distinct rainfall quantity RII became significantly negative (Figure 2a,b). The highest two rainfall years were statistically indistinguishable from each other but had significantly higher RII biomass values than the other 2 years (Figure 2b). Although the lowest rainfall year showed overall negative RII values, there were plots in the north and west aspects under some *Retama* with positive RII values.

**Figure 3:**
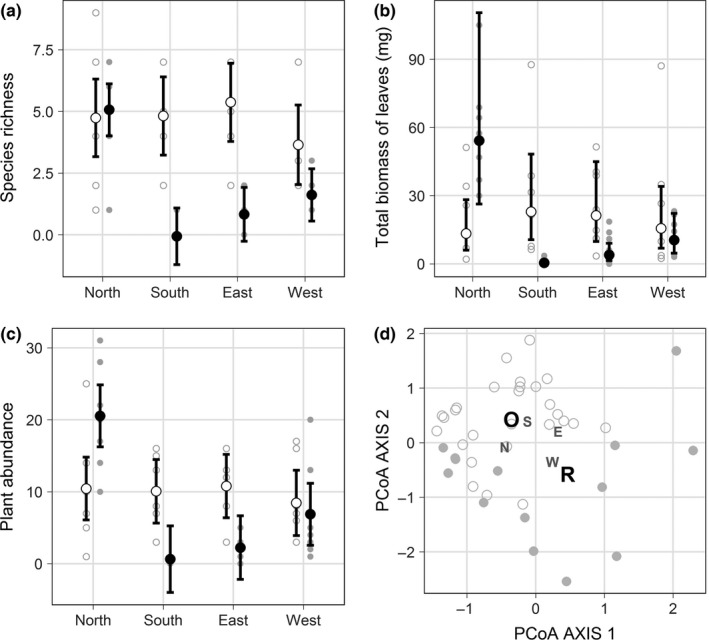
Plant richness, abundance, and composition under and away from *Retama*. (a) Plant species richness under (● closed circles) and outside (○ open circles) *Retama* in 2012, a year with below‐average rainfall. Richness was always higher outside *Retama* except in the north aspect of the shrub. Black points represent model estimates (95% CI), and gray points represent plot‐level observations. (b) Total biomass of leaves showed a similar pattern as richness with only the north aspect under *Retama* having higher biomass than outside *Retama* plots. The data were log‐transformed after adding 1 to meet assumptions of normality and back‐transformed to the normal scale in the figure. (c) Plant abundance patterns mirrored richness and total leaf biomass. (d) Plots under and outside *Retama* were composed of distinct plant species with few species shared. Points represent plots under *Retama* (closed circles) and in open locations (open circles) for each aspect, and the text represents the centroids of the location (“O” = open and “R” = *Retama*) and aspect (“N” = north, “E” = east, “W” = west, and “S” = south) factors from the constrained analysis of principal coordinates

In our spatial analysis of the lowest rainfall year, open plots around *Retama* had more species (mean richness in open plots = 5, 95% CI: 4–6) than plots under *Retama* (mean richness in plots under *Retama* = 2, 95% CI: 1–3; Table S3). Differences in species richness among aspects in open plots were statistically indistinguishable, while species richness under *Retama* varied from a minimum in the south (mean richness = 0, 95% CI: −1 to 1; Figure 3a) to a maximum in the north plots (mean richness = 5, 95% CI: 4–6; Figure 3a). Species richness in north aspect plots under *Retama* was statistically indistinguishable from that of open plots. Patterns of total leaf biomass mirrored species richness with all open plots having statistically indistinguishable leaf biomass, while *Retama* plots had highly variable leaf biomass with a low in the south (0.4 mg, 95% CI: 0.4–1.9) and a high in the north (54.2 mg, 95% CI: 26.3–110.5; Figure 3b).

Plant abundance also showed a similar pattern to that of species richness whereby open plots consistently had similar plant abundance (estimated abundance in open plots = 10 plants, 95% CI: 7–13; Table S3). In contrast, plots under *Retama* were significantly different among aspects with plant abundance in the south and east aspect being statistically indistinguishable from zero and the north aspect having significantly higher plant abundance than all other aspects (estimated abundance in the north aspect under *Retama* = 21 plants, 95% CI: 16–26; Figure 3c). Furthermore, the north aspect under *Retama* had significantly more plants than plots in open locations.

The composition of species was significantly different between plots under *Retama* and open plots (F_1,41_ = 3.5, *p *=* *.002; Figures 3d and S3). The interaction term of the constraining matrix was also significant (F_3,41_ = 1.8, *p *=* *0.01; Figure 3d) indicating that aspects under *Retama* also had significantly different compositions. Combined, location (i.e., under *Retama* and open) and aspect significantly explained 23.1% of the variation in plant community composition.

## 4 Discussion

Our combined assessment of temporal shifts in facilitation under *Retama* across years of variable rainfall and detailed spatial analyses of plant communities during a low rainfall year indicate that facilitation—as measured by species richness and community productivity—can in fact give way to competition or biotic filtering under severe resource limitation. However, positive effects in the lowest rainfall year were still present in the north aspect under most *Retama* shrubs, while the east, south, and west aspects had fewer species, reduced biomass, and lower plant abundance than plots away from the effects of *Retama*. Our results highlight two important points. (1) The shift from positive to negative effects was not a linear response to water availability but instead indicated a rainfall threshold below which competition or interference occurred, which supports previous work on facilitation in semiarid and desert plants (Madrigal‐González, Kelt, Meserve, Squeo, & Gutiérrez, 2016; Tielbörger & Kadmon, 2000). (2) Although overall negative effects of nurse plants may prevail under extreme resource limitation, we propose that spatial differences in the microhabitats under nurse plants provide a range of processes from facilitation to competition and interference. This spatial heterogeneity beneath nurse plants provides microrefugia for some species that depend on biotic modifications of the habitat by nurse plants, as evidenced by the unique composition of species in the north aspect beneath *Retama* (Figure 3d).

### 4.1 Response of facilitation to rainfall

In support of the hypothesis that facilitation shifts to competition under severe resource limitation (Michalet et al., 2014, 2015), RII for species richness and community productivity shifted to negative between 70 and 120 mm, while nearly universal positive effects were found above 120 mm. Our results directly support the conceptual model proposed by Tielbörger and Kadmon (2000) whereby under nurse plants water limitation occurs earlier than in open locations and beneficial effects such as improved nutrients or shading are overshadowed by competition for water. We propose two hypotheses to explain this threshold effect: (1) Competition for water under *Retama* is higher than in open locations during severely low rainfall years (Butterfield et al., 2016), or (2) species adapted to live under *Retama* have less tolerance to severe drought (Tielbörger & Kadmon, 2000). However, additional biotic and abiotic variables interact with rainfall to alter this threshold (Butterfield et al., 2010). For example, larger *Retama* plants increase niche space and microsite variability thereby often supporting higher diversity and productivity than smaller *Retama* (Pugnaire et al., 1996; Schöb et al., 2013) and delaying the effects of water limitation through increased shading. Differences among sampling designs and other climatic variables from the multiple years may have affected the results, but these artifacts were hopefully controlled for with the use of RII. Regardless, hotter and drier climatic conditions are expected in many Mediterranean ecosystems, which will continue to promote decreases in species richness and productivity.

### 4.2 Spatial differences in diversity, productivity, and composition

Overall plots under *Retama* failed to facilitate more diverse and productive understory communities during a severely low rainfall year, and the near absence of plants in the south and the east aspects indicates that *Retama* had a negative effect on community diversity and productivity (Michalet et al., 2015), further supporting the argument that facilitation gives way to competition under severe water limitation (Michalet et al., 2014; Tielbörger & Kadmon, 2000). In contrast to the other aspects, the north maintained species richness and community productivity equal to or above that of the open plots. This difference between north and south may be explained by plant shading (Table S1) that reduced evapotranspiration and heat stress in the north but not in the south (Butterfield et al., 2010; López‐Pintor et al., 2006) rather than by rainfall interception as suggested by Tielbörger and Kadmon (2000). Thus, competition for water alone does not explain the lack of facilitation effects under reduced rainfall because these negative effects were ameliorated by reduced heat stress in the north. These results prove a failure of facilitation to maintain species that require obligate facilitation—except in the north aspect—as water became severely limited.

The distinct species composition between under *Retama* and open locations (Figure 3d) indicates a filtering of species that was likely constraining species intolerant to the hot and dry conditions in the open locations—that is, cases of obligatory facilitation (Bertness & Callaway, 1994; Kraft et al., 2015; Madrigal‐González et al., 2016; Pugnaire et al., 1996; Schöb, Butterfield, & Pugnaire, 2012). This environmental filter may also have been acting on the community in the east, south, and west aspects under *Retama* under reduced water availability, as evidenced by the reduction in species richness and plant abundance. Therefore, *Retama* no longer provided the conditions required by the obligatory beneficiary species. Surprisingly, species commonly found in open locations did not fill the empty niche left by the absence of beneficiary species in the east, south, and west (Madrigal‐González et al., 2016). The lack of open‐location species under *Retama* implies an additional filter (either biotic or abiotic) that constrained these species from establishing beneath the nurse plant, as the species composition of the seed bank is similar throughout the area (Pugnaire & Lázaro, 2000). The reduction of light from shading may be one contributing factor preventing the establishment of open‐location species under the *Retama* (Moro et al., 1997). Although we cannot explicitly distinguish the mechanisms that are inhibiting the species from establishing in either direction (i.e., open into under *Retama* or under *Retama* into open), it is clear that during severe water limitation species normally buffered by *Retama* from the hot and dry conditions only predominate in the north aspect.

## 5 Conclusions

Our results indicate that the effect of *Retama* nurse plants on herbaceous communities shifts from facilitative to competitive or interfering with decreasing water availability in support of the recent modifications to the stress‐gradient hypothesis. Specifically, the balance between positive and negative effects by the nurse plant on the beneficiary community was spatially explicit shifting to competition in the east and the south and partially in the west—where diversity and productivity was lower under *Retama* relative to open locations. However, the north aspect under *Retama* maintained more species and plants and supported a distinct community composition relative to open locations. Because nurse plants provide refugia for distinct species in these semiarid ecosystems, a climate that is changing to hotter and drier conditions and promoting shifts from positive to negative interactions will hinder the persistence of species that depend on nurse shrubs and in turn put biodiversity in these important ecosystems at risk.

## Data accessibility

All data used in this manuscript will be archived in Dryad.

## Conflict of interest

None declared.

## Author contributions

MOB analyzed and wrote the manuscript and compiled the data for the meta‐analysis. FIP contributed to the writing and conceptual development of the manuscript. CA and SRE provided data for the meta‐analysis and revisions to the manuscript. CS conceived the experiment, performed the data collection in 2012, contributed to the analysis, and cowrote the manuscript.
