# Supplementary material for: The shift from plant–plant facilitation to competition under severe water deficit is spatially explicit
Source: Ecol Evol. 2017 Mar 12;7(7):2441–8. doi: 10.1002/ece3.2875 (PMC5383484; doi:10.1002/ece3.2875)
Supplement: Supplementary file 1 [file ECE3-7-2441-s001.docx]

**Supporting information**

**Table S1: Environmental variables from four aspects under *Retama*.**

**Table S2: ANOVA of RII response to rainfall.**

**Table S3: ANOVA of richness and productivity.**

**Fig S1: Diagram of plot design.**

**Fig S2: Relationship between biomass of largest leaf and total plant biomass.**

**Fig S3. Relative abundance of each species in *Retama* plots.**

**Appendix S1. Description of trait analysis.**

**Table S4: ANOVA of trait range and dispersion.**

**Fig S4: Trait-based assessment of niche space.**

**The shift from plant–plant facilitation to competition under severe water deficit is spatially explicit**

Michael J. O’Brien^1*^, Francisco I. Pugnaire^1^, Cristina Armas^1^, Susana Rodríguez-Echeverría^2^ and Christian Schöb^3*^

*^1^Estación Experimental de Zonas Áridas, Consejo Superior de Investigaciones Científicas, Carretera de Sacramento s/n, E-04120 La Cañada, Almería, Spain*

*^2^Centre for Functional Ecology, Department of Life Sciences, University of Coimbra, Calçada Martim de Freitas, 3000-456 Coimbra, Portugal*

*^3^Department of Evolutionary Biology and Environmental Studies, University of Zurich, Winterthurerstrasse 190, CH-8057 Zurich, Switzerland*

*^*^corresponding authors*: mikey.j.obrien@gmail.com and christian.schoeb@ieu.uzh.ch

**Table S1.** Mean environmental variables (SE) measured in each aspect under *Retama* *sphaerocarpa* during sampling in 2012 (PAR is photosynthetically active radiation).

| **Aspect** | **Soil temperature**  **(° C)** | **Air temperature**  **(° C)** | **Soil depth**  **(mm)** | **PAR**  **(µmol m^-2^ s^-1^)** |
| --- | --- | --- | --- | --- |
| North | 16.2 (0.3) | 22.8 (0.2) | 116.7 (5.4) | 608 (46) |
| East | 22.8 (0.3) | 24.6 (0.2) | 100.4 (6.6) | 859 (83) |
| South | 26.8 (0.9) | 25.9 (0.3) | 113.0 (6.4) | 1228 (120) |
| West | 18.0 (0.5) | 23.1 (0.4) | 108.9 (6.9) | 701 (65) |
| Open | 29.8 (0.2) | 26.2 (0.2) | 106.4 (3.2) | 1399 (22) |

**Table S2.** The ANOVA tables from the linear mixed-effects model of RII for (A) species richness and (B) productivity.

| **Source of variation** | **d.f.** | **denominator d.f.** | **F** |
| --- | --- | --- | --- |
| **A** |  |  |  |
| Total rainfall (continuous) | 1 | 66.6 | 10.7******* |
| Total rainfall (factor) | 2 | 15.0 | 32.0******* |
| **Variance components** | **Var.** | **SE** |  |
| Year:experiment:sample | 3E-03 | 1E-03 |  |
| Variance rain 2012 | 2E-01 | 5E-02 |  |
| Variance rain 2015 | 2E-02 | 1E-02 |  |
| Variance rain 2009 | 3E-07 | NA |  |
| Variance rain 2010 | 3E-02 | 6E-03 |  |
| **B** |  |  |  |
| Total rainfall (continuous) | 1.0 | 45.7 | 37.1******* |
| Total rainfall (factor) | 2.0 | 14.5 | 19.6******* |
| **Variance components** | **Var.** | **SE** |  |
| Year:experiment:sample | 0.02 | 0.01 |  |
| Variance rain 2012 | 0.30 | 0.08 |  |
| Variance rain 2015 | 0.05 | 0.04 |  |
| Variance rain 2009 | 0.01 | 0.01 |  |
| Variance rain 2010 | 0.01 | 0.00 |  |
|  |  |  |  |

d.f., degrees of freedom; denominator d.f., denominator degrees of freedom,

F, conditional F-statistic; Var., variance component estimate and SE, standard errors for random effects; *******P < 0.001

**Table S3.** The ANOVA tables from the linear mixed-effects model of (A) species richness, (B) sum of the biomass of the largest leaves (log of biomass plus one) and (C) plant abundance.

| **Source of variation** | **d.f.** | **denominator d.f.** | **F** |
| --- | --- | --- | --- |
| **A** |  |  |  |
| Soil depth | 1 | 13.9 | 1.4 |
| Soil temperature | 1 | 15.9 | 33.6*** |
| Location | 1 | 23.4 | 21.4*** |
| Aspect | 3 | 16.6 | 10.1*** |
| Location x aspect | 3 | 19.9 | 8.2*** |
| **Variance components** | **Var.** | **SE** |  |
| Retama plant | -0.1 | 0.3 |  |
| Retama:location | -0.2 | 0.3 |  |
| Retama:aspect | 1.0 | 0.8 |  |
| VarianceOpen | 3.6 | 1.2 |  |
| VarianceRetama | 1.2 | 0.8 |  |
| **B** |  |  |  |
| Soil depth | 1 | 43.2 | 0.3 |
| Soil temperature | 1 | 24.1 | 7.5* |
| Location | 1 | 14.3 | 1.5 |
| Aspect | 3 | 17.2 | 8.2** |
| Location x aspect | 3 | 18.2 | 15.1*** |
| **Variance components** | **Var.** | **SE** |  |
| Retama plant | -0.01 | 0.13 |  |
| Retama:location | 0.12 | 0.19 |  |
| Retama:aspect | 0.02 | 0.18 |  |
| Residual | 0.70 | 0.24 |  |
| **C** |  |  |  |
| Soil depth | 1 | 29.5 | 0.59 |
| Soil temperature | 1 | 25.9 | 2.64 |
| Location | 1 | 13.4 | 0.26 |
| Aspect | 3 | 17.5 | 12.3*** |
| Location x aspect | 3 | 18.3 | 7.55** |
| **Variance components** | **Var.** | **SE** |  |
| Retama plant | -2.1 | 4.3 |  |
| Retama:location | 3.1 | 7.8 |  |
| Retama:aspect | -5.8 | 7.4 |  |
| Residual | 36.0 | 12.2 |  |

d.f., degrees of freedom; denominator d.f., denominator degrees of freedom,

F, conditional F-statistic; Var., variance component estimate and SE, standard errors for random effects; *****P < 0.05, ******P < 0.01, *******P < 0.001


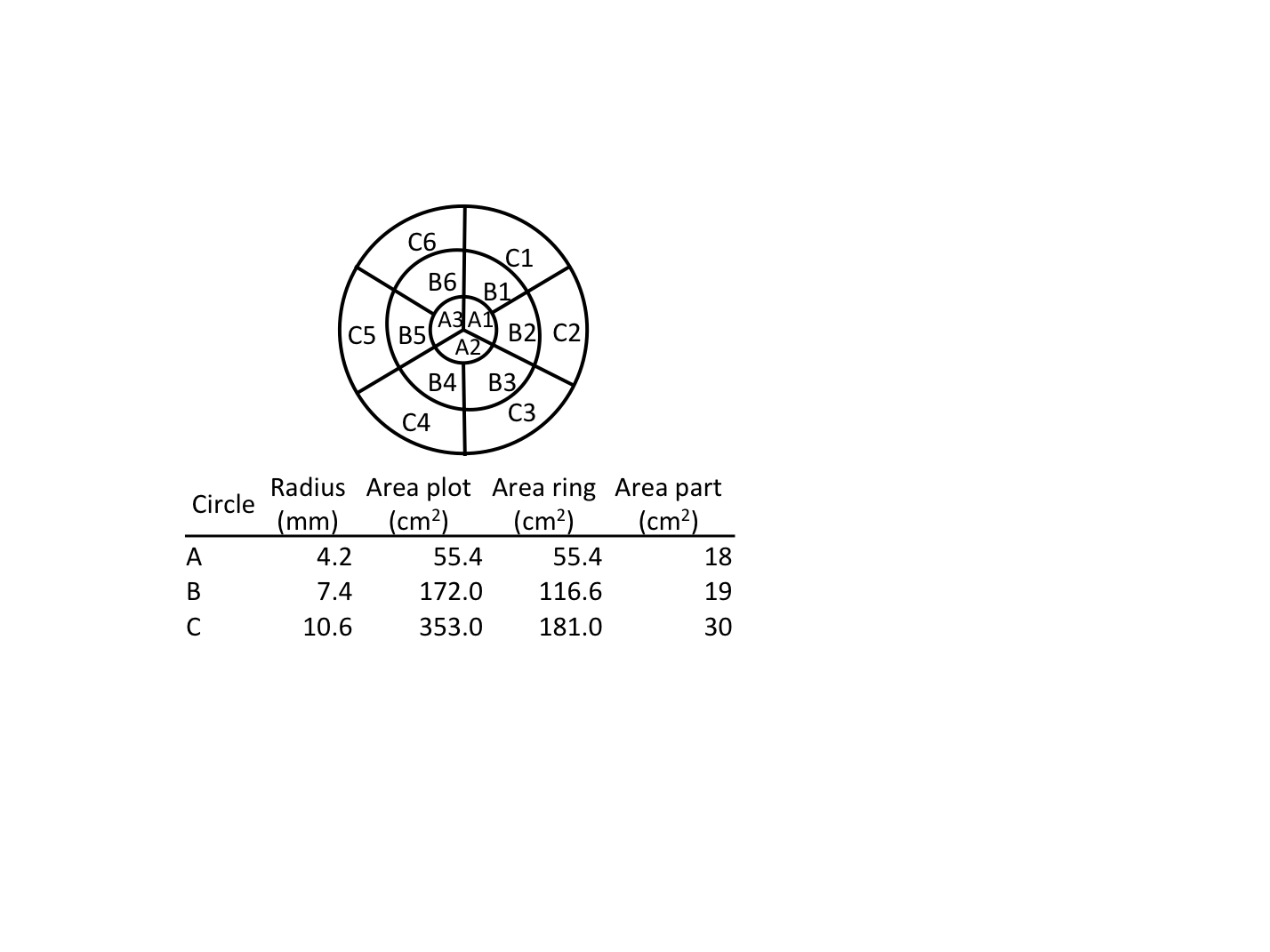


**Fig. S1 Design of plot layout.** Circular sampling grid used for all plots to study plant species composition, plant traits and abiotic conditions. All abiotic measurements were conducted in each part of the plot, identified by a unique code consisting of the ring (A-C) and the part within each ring (1-6), whereas plant traits were measured for all individuals occurring in all parts of the plot.

**
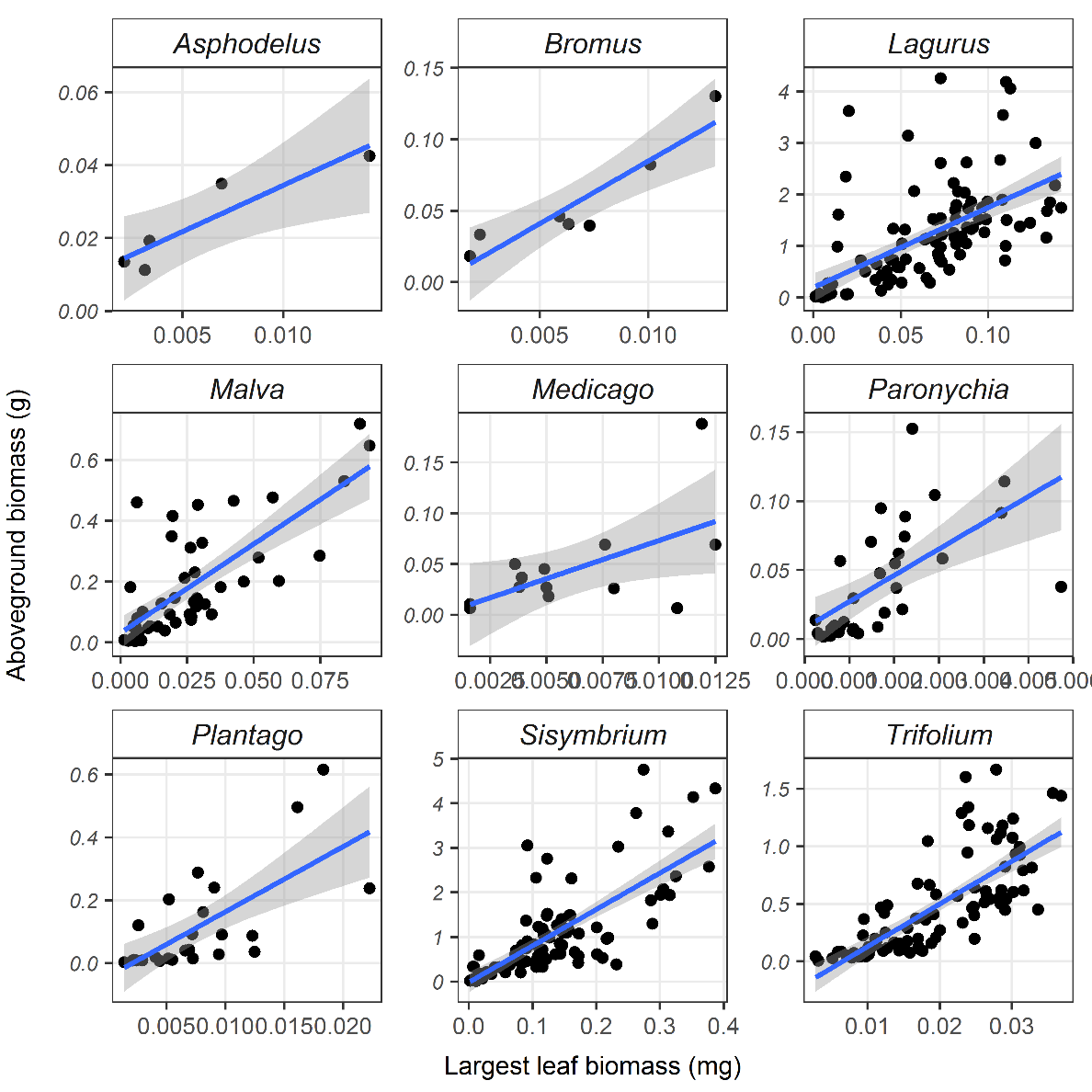
**

**Fig. S2 Linear regression between biomass of largest leaf and total aboveground biomass.** Linear regressions of aboveground biomass (95% CI) as a function of largest-leaf biomass for nine common species found in the ecosystem. The data were collected from three studies (two *in situ* and one greenhouse). All regressions show a significant positive relationship (*Asphodelus fistulosus*, F_1,3_ = 17.1, p = 0.03; *Bromus rubens*, F_1,5_ = 29.5, p = 0.003; *Lagurus ovatus*, F_1,104_ = 65.6, p < 0.0001; *Malva parviflora*, F_1,46_ = 60.7, p < 0.0001; *Medicago minima*, F_1,11_ = 5.5, p = 0.04; *Paronychia argentea*, F_1,30_ = 18.1, p = 0.0002; *Plantago albicans*, F_1,23_ = 21.0, p = 0.0001; *Sisymbrium erysimoides*, F_1,94_ = 126.8, p < 0.0001; *Trifolium stellatum*, F_1,93_ = 123.5, p < 0.0001).

**
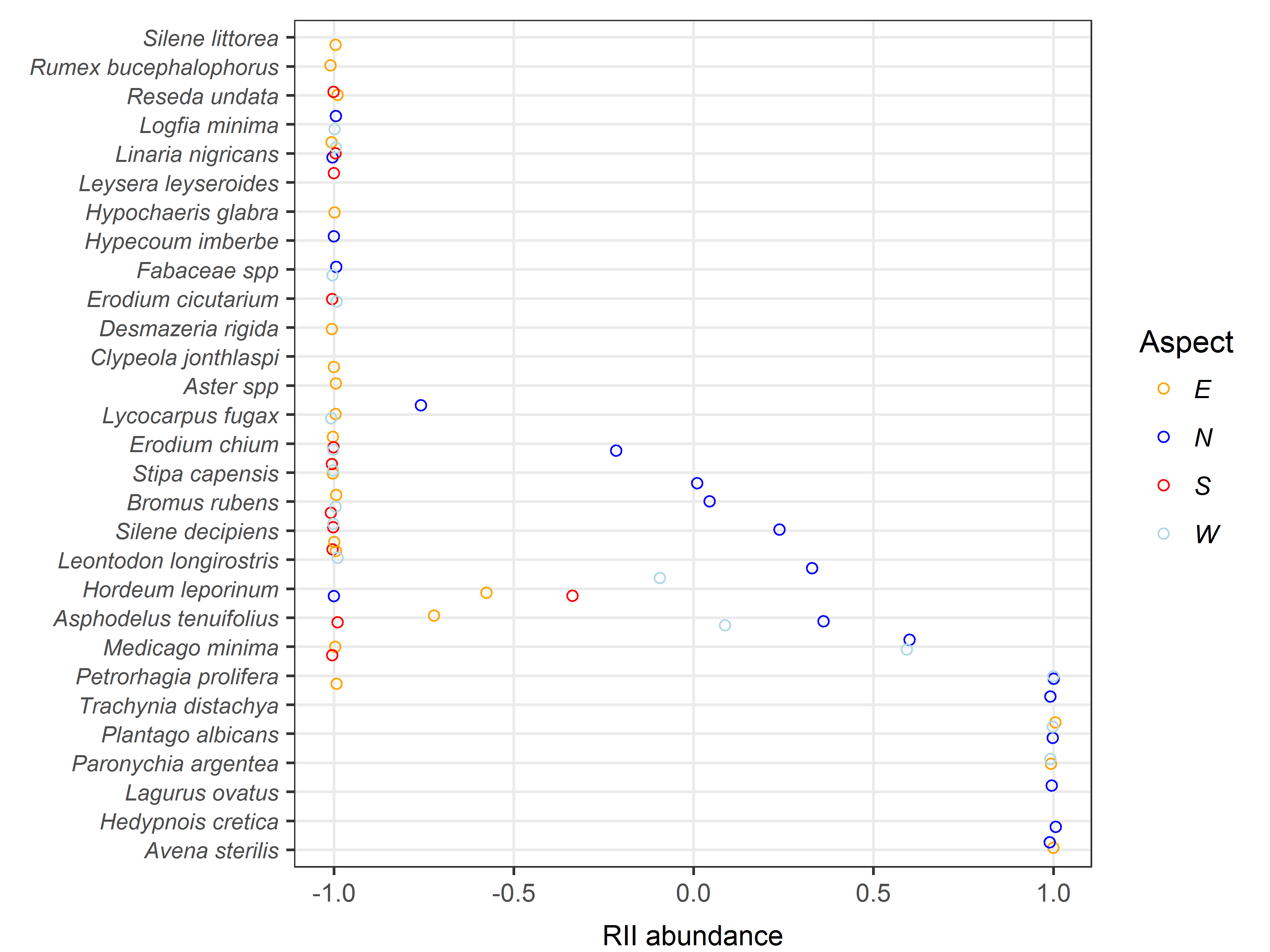
**

**Fig. S3** **Relative abundance of each species in the four aspects under *Retama*.** The relative interaction index for each species under *Retama*. The species are ordered from low to high relative abundance in *Retama* plots.

**Appendix S1. Description of trait collection and analysis and results.**

**Collection and analysis of trait data**

For each individual (486 individuals in total), we measured plant height and took the largest healthy leaf (including the petiole). Collected leaves were rehydrated, scanned for leaf area, weighed and finally dried to constant weight at 80 °C for determination of leaf dry mass. Leaf dry matter content was then calculated as leaf dry mass divided by water-saturated fresh mass.

We performed trait-based calculations of niche breadth (i.e. the range of trait values) and niche differentiation (i.e. trait dispersion quantified as the coefficient of variation of the successive trait values) in each plot as described by Kraft and Ackerly (2010 for leaf dry matter content (LDMC) and maximum plant height (the height reached at the end of the growing season). We analyzed these niche metrics as a function of location (a fixed factor with 2 levels; under *Retama* and open), aspect (a fixed factor with 4 levels; north, east, south and west) and their interaction with random effects for *Retama* plant, location nested within *Retama* plant and aspect nested within *Retama* plant. The south aspect was removed from this analysis because only two plants were found in total in this aspect under *Retama*.

**Table S4.** The ANOVA tables from the linear mixed-effects model of (A) leaf dry matter content range, (B) leaf dry matter content dispersion, (C) maximum height range and (D) maximum height dispersion.

| **Source of variation** | **d.f.** | **denominator d.f.** | **F** |
| --- | --- | --- | --- |
| **A** |  |  |  |
| Location | 1 | 3.9 | 30.28** |
| Aspect | 2 | 11.1 | 1.08 |
| Location x aspect | 2 | 11.6 | 3.30^†^ |
| **Variance components** | **Var.** | **SE** |  |
| Retama plant | 266.88 | 1795.33 |  |
| Retama:location | -3503.99 | 1817.52 |  |
| Retama :aspect | 3037.35 | 4533.67 |  |
| Residual | 12065.14 | 5151.38 |  |
| **B** |  |  |  |
| Location | 1 | 5.9 | 0.09 |
| Aspect | 2 | 7.5 | 18.63** |
| Location x aspect | 2 | 12.3 | 1.27 |
| **Variance components** | **Var.** | **SE** |  |
| Retama plant | 0.04 | 0.03 |  |
| Retama:location | -0.04 | 0.05 |  |
| Retama:aspect | -0.13 | 0.06 |  |
| Residual | 0.27 | 0.11 |  |
| **C** |  |  |  |
| Location | 1 | 6.1 | 1.123 |
| Aspect | 2 | 11 | 3.568^†^ |
| Location x aspect | 2 | 12.1 | 1.696 |
| **Variance components** | **Var.** | **SE** |  |
| Retama plant | -24.93 | 100.36 |  |
| Retama:location | -38.80 | 182.32 |  |
| Retama:aspect | -192.25 | 204.14 |  |
| Residual | 835.94 | 348.81 |  |
| **D** |  |  |  |
| Location | 1 | 5.6 | 1.26 |
| Aspect | 2 | 12 | 8.71** |
| Location x aspect | 2 | 10.4 | 1.06 |
| **Variance components** | **Var.** | **SE** |  |
| Retama plant | -0.01 | 0.02 |  |
| Retama:location | 0.01 | 0.04 |  |
| Retama:aspect | 0.01 | 0.04 |  |
| Residual | 0.10 | 0.05 |  |
|  |  |  |  |

d.f., degrees of freedom; denominator d.f., denominator degrees of freedom,

F, conditional F-statistic; Var., variance component estimate and SE, standard errors for random effects; ^†^P<0.1, *****P < 0.05, ******P < 0.01, *******P < 0.001


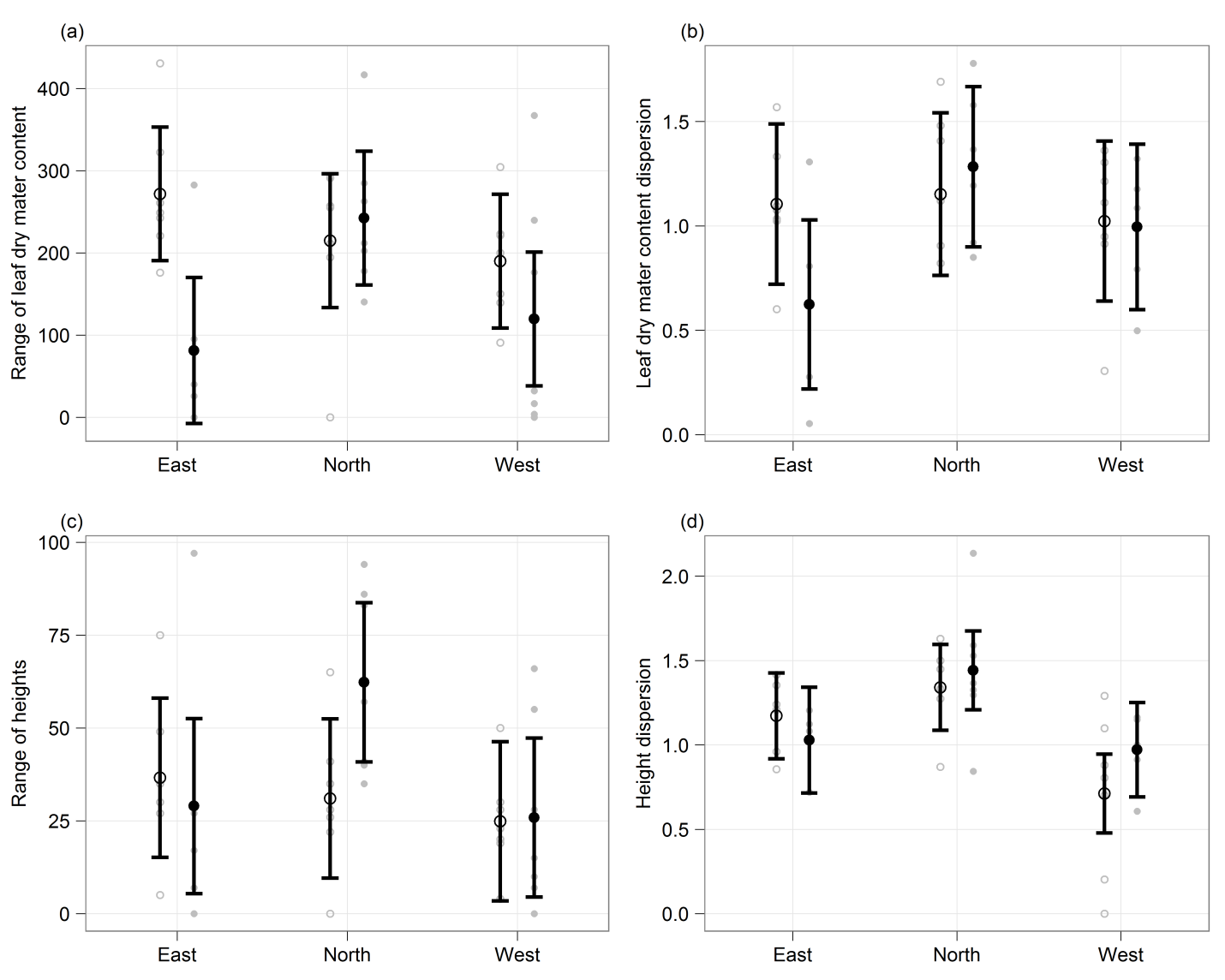


**Fig. S4 Trait-based assessment of niche space.** (a) Range of leaf dry matter content (LDMC) under (● closed-circles) and outside (○ open-circles) *Retama*. Range of LDMC was similar beneath and outside *Retama* except in the east aspect of the shrub. Black points represent model estimates (95% CI) and grey points represent plot level observations. (b) LDMC dispersion was similar among locations and aspects. (c) Range of plant height was similar beneath and outside *Retama* except in the north aspect of the shrub. (d) Height dispersion was similar among locations and aspects. The south aspect was removed due to lack of herbaceous plants.
